# Drinking During Adolescence

**Published:** 1996

**Authors:** Laurie Chassin, Christian DeLucia

**Affiliations:** Laurie Chassin, Ph.D., is professor of psychology and Christian DeLucia is a graduate research assistant in the Psychology Department, Arizona State University, Tempe, Arizona

**Keywords:** adolescent, adolescence, AOD consumption, AOD associated consequences, risk factors, sociocultural AODC (causes of AOD use, abuse, and dependence), family as an AODC, peer relations, personality trait, AOD use initiation

## Abstract

For many people, experience with drinking alcohol begins in adolescence. Yet for some youth, such early experimentation can spiral into problematic drinking patterns. Research has associated a variety of serious health risks with adolescent drinking, including the three leading causes of death among this age group (i.e., unintentional injuries, homicide, and suicide) as well as unsafe sexual behavior. Although alcohol abuse and dependence are not often clinically diagnosed among youth of high school age and younger, it is possible that applying adult diagnostic criteria does not fully capture the extent of adolescent problem drinking. Risk factors for adolescent drinking encompass sociocultural factors, such as regulation of alcohol availability; parental behavior and drinking patterns; the influence and drinking habits of siblings and peers; personality traits, particularly those indicating low self-regulation; and positive beliefs about alcohol’s effects. More research is necessary, however, to distinguish the factors that can predict adolescent problem drinking from those that predict consumption in general.

Researchers typically consider adolescence to be the developmental period during which youth are most at risk for initiating alcohol use. Many developmental theorists view adolescence as occurring from approximately age 10 (i.e., the beginning of pubertal development) through age 25 (i.e., when adult roles are established) (Feldman and Elliott 1990). This article, however, applies a more restrictive definition and primarily considers alcohol consumption from early adolescence through the high school years (i.e., approximately ages 10 to 18).

Prevalence data suggest that most adolescents report some exposure to alcohol use and that use increases with age (Johnston et al. 1995). In terms of current consumption, recent national student survey data (see figure 1) show that 25 percent of 8th graders and 50 percent of 12th graders report consuming alcohol within the past month (Johnston et al. 1995). Moreover, a substantial proportion of those drinkers consume heavily:^1^ 15 percent of the 8th graders and 28 percent of the 12th graders report having five or more drinks in a row in the past 2 weeks, and just under 3 percent of the 12th graders report daily use. National surveys have found some gradual declines in adolescent drinking since the peaks of the early 1980’s, but the most recent trends, which show some small (i.e., not statistically significant) increases (Johnston et al. 1995), are less clear.

The prevalence of adolescent drinking also varies demographically. Boys report more heavy drinking than do girls, and among high school seniors, white adolescents report more heavy drinking than do Hispanic or African-American adolescents. For example, 1994 national data for 12th graders (see figure 2) found that 32 percent of non-Hispanic Caucasians, 24 percent of Hispanics, and 14 percent of African-Americans reported consuming five or more drinks in a row in the past 2 weeks (Johnston et al. 1995). However, because these ethnic differences are less apparent at earlier grades, they could be caused partially by ethnic differences in school dropout rates (Johnston et al. 1995). (School-based surveys collect data on current students only; thus, these surveys cannot determine the alcohol consumption patterns of same-age school dropouts. To the degree that dropout rates vary by ethnicity, the survey results will be skewed.) Inconsistent definitions, as well as inter- and intracultural diversity, complicate comparisons among ethnic groups. In general, however, compared with other ethnic groups, Native American adolescents typically report the highest consumption rates, and Asian-American youth report the lowest (Johnstone 1994).

## Diagnosis of Alcohol Abuse and Dependence Among Adolescents

As with adult alcohol use, an examination of adolescent drinking patterns and problems requires consideration of (1) the quantity and frequency of consumption, (2) alcohol-related negative life consequences, and (3) alcohol-dependence symptoms (Bailey and Rachal 1993). Beyond the examination of simple consumption patterns, however, a striking lack of empirical work exists on the prevalence of clinical alcohol abuse or dependence^2^ among adolescents of high school age or younger. This void likely is attributable to the low prevalence of alcohol abuse and dependence diagnoses in early and middle adolescence compared with the dramatic increase in alcohol problems after the high school years. For example, Cohen and colleagues (1993) found prevalence rates^3^ of 4 percent or less among adolescents younger than age 16. In contrast, prevalence rates of 8.9 percent and 20.3 percent, respectively, were found for females and males ages 17 to 20 (Cohen et al. 1993).

Simply applying adult diagnostic criteria, however, may not be the best way to describe the drinking patterns and problems that occur during early and middle adolescence. Using other measures, the discrepancy between prevalence rates for younger and older adolescents could diminish. For example, Martin and colleagues (1995) found that common adult symptoms of alcohol abuse and dependence, such as medical problems and alcohol withdrawal symptoms, rarely occurred in adolescents. In addition, many adolescents who did not have an alcohol-dependence diagnosis reported a marked increase in the amount of alcohol needed to attain a desired effect (i.e., tolerance). In fact, Martin and colleagues (1995) suggest that a marked increase in consumption may be a normative feature of adolescent drinking rather than a phenomenon linked specifically to alcohol dependence. Research is now under way to refine methods for diagnosing clinical alcohol abuse and dependence in adolescents (Martin et al. 1995).

## Consequences of Adolescent Drinking

Adolescent alcohol use (particularly heavy use) is associated with many negative outcomes (see table 1). Although medical consequences of alcohol abuse in adolescents are rarely studied, a few studies have produced evidence that describes such effects. For example, adolescent alcohol abusers show elevations in liver enzymes (Arria et al. 1995), an early indicator of liver damage. These adolescents also demonstrate higher rates of multiple drug use (Arria et al. 1995) and poorer language function than do adolescents without alcohol abuse or dependence diagnoses (Moss et al. 1994). However, they do not show signs of brain damage on neuropsychological tests (Moss et al. 1994).

Perhaps of even greater public health significance is the fact that adolescent alcohol consumption is correlated with the three leading causes of death in this age group: unintentional injuries, homicide, and suicide (U.S. Department of Health and Human Services [USDHHS] 1991). More than one-half of all fatal motor vehicle crashes among 15- to 24-year-olds involve alcohol, and approximately one-half of all homicides in this age group are associated with alcohol use (USDHHS 1991). Moreover, the percentage of intoxicated drivers involved in fatal crashes is higher at younger ages, reaching a peak among young adults (i.e., 34 percent for drivers ages 21 to 24 in 1991), then declining among older adults (i.e., 16 percent for drivers ages 45 to 64 in 1991) (National Center for Statistics and Analysis 1992). National data also suggest that alcohol use was associated with suicidal thoughts and suicide attempts among the 8th and 10th graders who were surveyed (Windle et al. 1992).

The association between adolescent alcohol consumption and risky sexual behavior also is of public health importance. Adolescent alcohol use is associated with earlier initiation of sexual activity, more frequent sexual activity, and less frequent condom use (Cooper et al. 1994), all of which raise the risk for HIV infection and other sexually transmitted diseases. In addition, adolescents (particularly white adolescents) report riskier sexual behavior on occasions when they have used alcohol or other drugs (AOD’s) than on occasions when they have not (Cooper et al. 1994).

However, the association between adolescent drinking and these serious negative health risks does not imply a causal relationship. As other researchers have indicated (Donovan 1993; Leigh and Stall 1993), adolescent alcohol use is associated with personality characteristics such as impulsiveness and sensation seeking. Thus, it may be these underlying personality characteristics, rather than simply alcohol use, that increase the risk for traffic crashes, risky sexual behavior, violence, and suicide. A similar argument has been made concerning the relationship between alcohol use and other drug use. Alcohol is used at earlier ages than are other drugs, and alcohol use increases the risk for later use of illegal drugs (Yamaguchi and Kandel 1984). Early onset of alcohol use (i.e., before age 15) is associated with greater risk for other substance use and the development of later alcohol-related problems (Robins and Pryzbeck 1985). However, although alcohol, like cigarettes, may lead to other forms of substance use, this pattern does not imply that alcohol use “causes” such substance use.

In terms of psychosocial development, Baumrind and Moselle (1987) speculate that heavy AOD use in adolescence interferes with the development of emerging adolescent competencies, including social and coping skills. Because most adolescents report some alcohol use, however, these deficits may be limited to those adolescents who drink particularly heavily or frequently. In fact, in the general population, drinking in adolescence has been associated with enhanced social functioning, less loneliness, and more positive emotional states (i.e., positive affect) in early adulthood (Newcomb and Bentler 1988).

## Risk Factors for Adolescent Drinking

Risk factors for adolescent drinking can be organized into categories that include sociocultural, family, peer, and intrapersonal factors as well as factors related to adolescents’ beliefs about alcohol.

### Sociocultural Factors

One sociocultural factor affecting adolescents is their degree of access to alcohol. Although few studies have focused specifically on adolescents (Holder 1994), the existing research suggests that greater alcohol availability is associated with higher rates of drinking. In contrast, greater regulation of alcohol availability is associated with older ages of initiation, decreased consumption, and fewer alcohol-related problems (Single 1994). In particular, computer simulations indicate that policies raising the legal drinking age or increasing prices with alcohol taxes are associated with lower rates of adolescent alcohol consumption and reduced mortality from traffic crashes among youth ages 18 to 20 (Grossman et al. 1994). Research on other sociocultural factors, such as the impact of alcohol advertising and alcohol warning labels, is equivocal and has produced limited data on adolescents (MacKinnon 1995; Single 1994).

### Family Factors

Many theories (e.g., social control theory, social learning theory, and problem behavior theory) include a focus on family factors that influence adolescent alcohol use (Jacob and Leonard 1994). Empirical studies have produced fairly consistent support for these theories. Families in which parents use alcohol to excess, show high levels of antisocial behavior (including antisocial personality disorder^4^), or both are said to model alcohol-abusing behavior, a factor termed “family modeling.” These families are more likely to have adolescent children who use alcohol (see the article by Windle on parental alcoholism, pp. 181–184). Families in which parents provide low levels of social support, show little monitoring of their children’s behavior, use inconsistent discipline practices, and exhibit high levels of conflict and low levels of closeness—traits known as family socialization factors—also are more likely to have adolescent children who use alcohol (Barnes et al. 1986). Most empirical studies have been conducted with biological families, however, which may lead to overestimating the magnitude of such family influences, because the behavior of offspring reflects shared genes as well as shared environment (McGue et al. 1996). (In contrast, studies of adoptees can help isolate environmental factors from genetic ones.)

Potentially important components of family influence on adolescent drinking are the drinking behavior and social influence of siblings (Jacob and Leonard 1994). Siblings’ levels of alcohol consumption are correlated for both biological and adoptive siblings. These correlations are stronger for siblings who are close in age and of the same sex (McGue et al. 1996). Siblings may provide direct modeling influences as well as more indirect influences through exposure to a particular high-risk peer group (Rowe and Gulley 1992).

### Peer Factors

Peer drinking and peer acceptance of drinking (i.e., positive attitudinal tolerance of drinking) have been consistently associated with adolescent drinking, and adolescent drinking typically occurs in peer social contexts (Hughes et al. 1992; Margulies et al. 1977). Adolescents whose friends frequently drink are more likely to increase their own drinking over time, and adolescents who frequently drink are more likely to increase their affiliations with alcohol-using peers (Curran et al. in press). Thus, adolescents who drink are more likely to select friends who drink, and those friends in turn influence adolescents’ drinking.

In understanding adolescents’ risk for drinking, one must consider what leads adolescents to affiliate with alcohol-using peers. Some research suggests that poor parenting practices create early childhood deficits in social skills and self-regulation, particularly with regard to aggressive behavior, which result in rejection from mainstream peer groups (Brown et al. 1993; Patterson et al. 1989). Children who are rejected from these mainstream peer groups then affiliate with deviant peers; in turn, participation in deviant peer networks increases the risk for drinking and other forms of substance use (Kaplan 1980). This research demonstrates a link between the parenting factors and the peer factors that lead to adolescent drinking.

### Intrapersonal Factors

Adolescents who report high levels of alcohol consumption are characterized by a constellation of personality traits indicating low levels of self-regulation (see table 2). These adolescents are more likely to be aggressive and to have high attitudinal tolerance for deviant behavior, low value and expectations for academic success, and high levels of sensation seeking and impulsivity (Brook et al. 1992; Jessor and Jessor 1977). These characteristics also describe adolescents with clinical levels of alcohol abuse or dependence (Moss and Kirisci 1995).

The role of other intrapersonal factors is more controversial. For example, it is unknown whether intense emotional responses and a tendency to overreact (i.e., emotional reactivity) or negative emotional states, such as depression and anxiety, are linked to adolescent alcohol use. Some data link negative emotional states, particularly depression, to adolescent alcohol use (Colder and Chassin 1993; Hussong and Chassin 1994). However, studies over time that attempt to predict later alcohol use based on previously measured levels of negative emotional states do not always confirm this relationship (Chassin et al. 1996). Thus, it is unclear whether negative emotional states are a cause or a result of adolescent alcohol use, although depressive disorders (Deykin et al. 1992) and anxiety disorders (Clark et al. 1994) have been associated with clinical alcoholism in adolescence. Moreover, the combination of low self-regulation and high levels of negative emotional states (i.e., negative affect) may be associated particularly with adolescent alcohol use (Pandina et al. 1992).

The onset of adolescent alcohol use among middle school students also has been linked to low levels of self-esteem (Kaplan 1980). According to Kaplan’s self-derogation theory, adolescents who receive failure feedback from mainstream sources (e.g., peer rejection or poor school achievement) seek out deviant peer affiliations in order to increase their sense of self-worth. Although these deviant peer affiliations do raise self-esteem, they also raise risk for AOD use.

### Beliefs About Alcohol

Adolescents form beliefs about alcohol’s effects before actually engaging in alcohol consumption, and these expectations (i.e., alcohol expectancies) are related to their drinking behavior. For example, Christiansen and colleagues (1989) found that positive expectancies of alcohol predicted adolescents’ drinking behavior (and problem drinking) 12 months later. Moreover, among 12- to 14-year-old abstainers, adolescents who expected to gain greater social acceptance (i.e., social facilitation) as a result of drinking were more likely to begin to drink, and they increased their alcohol consumption at faster rates than did their same-age peers who did not show these expectancies (Smith et al. 1995). Alcohol expectancies are a potentially important risk factor for adolescents, because they may integrate adolescents’ knowledge about alcohol from sources such as media, peer, and family models as well as from their own experiences. Thus, many different influences can shape adolescents’ beliefs about alcohol, and these beliefs in turn influence adolescents’ drinking behavior.

## Summary and Conclusions

Understanding alcohol use in adolescence is critical, because during these years, many people initiate drinking, and early drinking problems can appear. Although some experimentation with drinking is virtually universal and normative in adolescence, alcohol use during this period also is linked to negative adolescent health outcomes, including unintentional injuries, homicide, suicide, and unsafe sexual practices. At a broad social level, adolescent drinking is related to alcohol availability as well as to laws, social norms, and prices regulating such availability. In addition, adolescents who drink alcohol are more likely to come from families in which parental drinking, sibling drinking, and lower levels of parental control and support occur. Youth who drink also are more likely to have friends who model and tolerate alcohol use, show lower levels of self-regulation, and have more positive expectancies about alcohol’s effects. Much of the research to date, however, has related these risk factors to the frequency or quantity of drinking rather than to alcohol abuse or dependence among adolescents. Thus, it has been difficult to distinguish predictive factors specific to adolescent problem drinking or clinical alcohol abuse or dependence from predictors of alcohol consumption in general. An understanding of the factors that determine which adolescents are particularly vulnerable to the negative effects of alcohol consumption is an important step for preventing alcohol-related problems among adolescents.

## Figures and Tables

**Figure 1:**
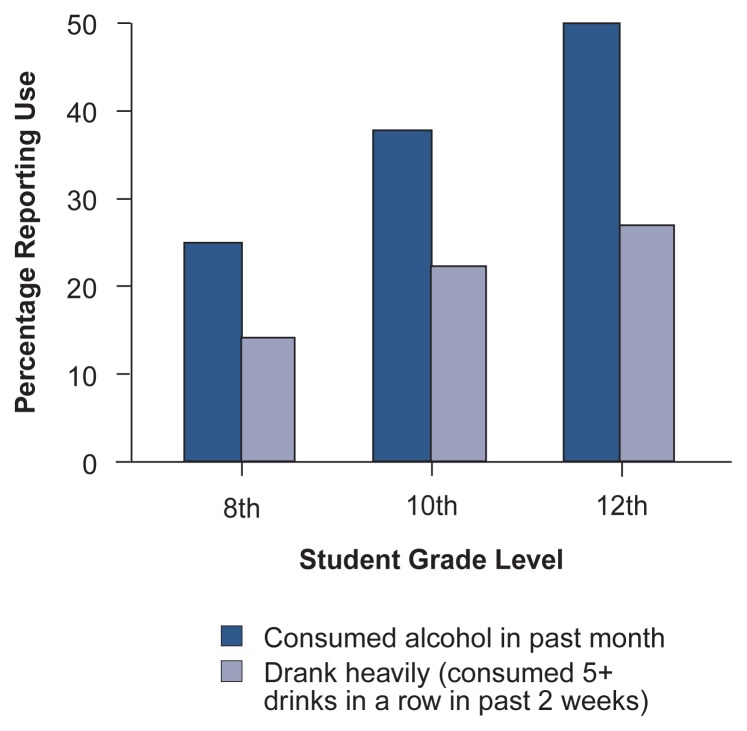
Past-month and heavy drinking among 8th, 10th, and 12th graders (1994). SOURCE: Johnston et al. 1995, p. 43.

**Figure 2:**
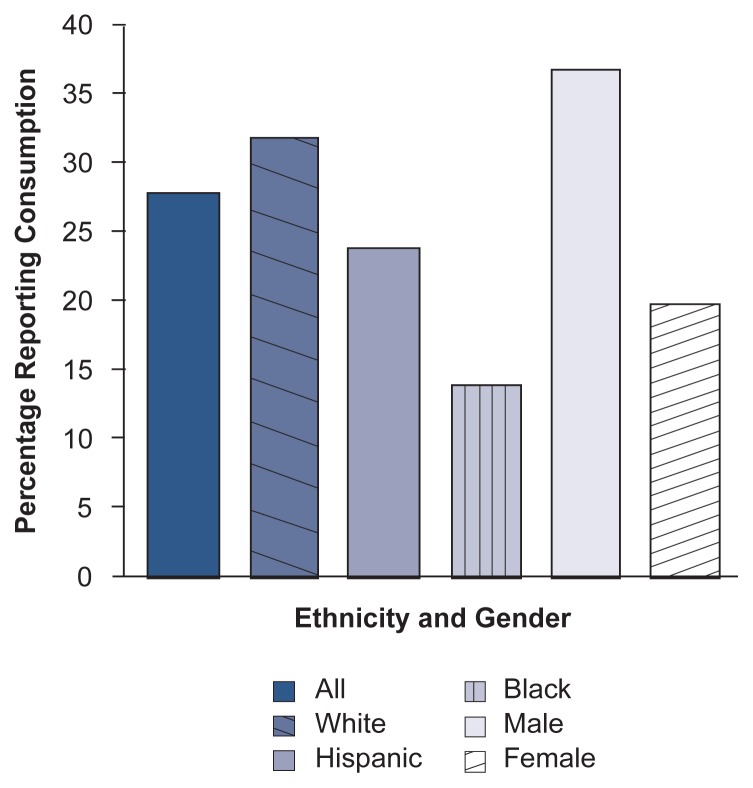
Demographic characteristics of 12th graders reporting consumption of 5+ drinks in a row in the past 2 weeks (1994). SOURCE: Johnston et al. 1995, p. 317.

**Table 1:** Negative Outcomes Associated With Adolescent Alcohol Use (Especially Heavy Use)

Health-Related Outcomes
Elevations in liver enzymes
Use of other drugs
Fatal motor vehicle crashes
Unintentional injuries
Homicide
Suicide
Early sexual activity
More frequent sexual activity
Less frequent condom use
**Psychosocial Outcomes**

Poorer language function
Interference with development of adolescent competencies
Interference with development of social and coping skills

**Table 2:** Personality Characteristics Associated With Adolescent Alcohol Use

Characteristics

Low self-regulation
Aggressiveness
Highly tolerant attitudes toward deviant behavior
Low value of and expectation for academic success
Sensation seeking
Impulsivity
Low self-esteem
**Possible Other Characteristics**

Negative emotional states (e.g., depression)
High emotional intensity
Low threshold for emotional response

## Footnotes

1 The term “heavy alcohol consumption” is not used consistently in the alcohol research literature, and the studies referred to in this article likewise define the concept in different ways. Unless otherwise specified, this article defines heavy consumption as having five or more drinks in a row within the past 2 weeks.

2 Definition of the terms “alcohol abuse” and “alcohol dependence” can vary in the alcoholism literature. Generally, however, a person clinically diagnosed with alcohol abuse or alcohol dependence must meet specific criteria (e.g., as defined in the American Psychiatric Association’s *Diagnostic and Statistical Manual of Mental Disorders, Fourth Edition*). These criteria include the experience of withdrawal symptoms and increased tolerance to the effects of alcohol as well as impaired control over drinking.

3 Using criteria from the American Psychiatric Association’s Diagnostic and Statistical Manual of Mental Disorders, Third Edition, Revised.

4 Antisocial personality disorder is a pattern of irresponsible and antisocial behavior beginning early in life and continuing through adulthood.

## References

[b1-arhw-20-3-175] Arria AM, Dohey MA, Mezzich AC, Bukstein OG, Van Thiel DH (1995). Self-reported health problems and physical symptomatology in adolescent alcohol abusers. Journal of Adolescent Health.

[b2-arhw-20-3-175] Bailey SL, Rachal JV (1993). Dimensions of adolescent problem drinking. Journal of Studies on Alcohol.

[b3-arhw-20-3-175] Barnes GM, Farrell MP, Cairns A (1986). Parental socialization factors and adolescent drinking behaviors. Journal of Marriage and the Family.

[b4-arhw-20-3-175] Baumrind D, Moselle KA (1987). A developmental perspective on adolescent drug abuse. Advances in Alcohol and Substance Abuse.

[b5-arhw-20-3-175] Brook JS, Cohen P, Whiteman M, Gordon AS, Glantz M, Pickens R (1992). Psychosocial risk factors in the transition from moderate to heavy use or abuse of drugs. Vulnerability to Drug Abuse.

[b6-arhw-20-3-175] Brown BB, Mounts N, Lamborn SD, Steinberg L (1993). Parenting practices and peer group affiliation in adolescence. Child Development.

[b7-arhw-20-3-175] Chassin L, Curran PJ, Hussong AM, Colder CR (1996). The relation of parent alcoholism to adolescent substance use: A longitudinal follow-up study. Journal of Abnormal Psychology.

[b8-arhw-20-3-175] Christiansen BA, Smith GT, Roehling PV, Goldman MS (1989). Using alcohol expectancies to predict adolescent drinking behavior after one year. Journal of Consulting and Clinical Psychology.

[b9-arhw-20-3-175] Clark DB, Jacob RG, Mezzich A (1994). Anxiety and conduct disorders in early onset alcoholism. Annals of the New York Academy of Sciences.

[b10-arhw-20-3-175] Cohen P, Cohen J, Kasen S, Velez CN, Hartmark C, Johnson J, Rojas M, Brook J, Streuning EL (1993). An epidemiological study of disorders in late childhood and adolescence—I. Age-and gender-specific prevalence. Journal of Child Psychology and Psychiatry and Allied Disciplines.

[b11-arhw-20-3-175] Colder CR, Chassin L (1993). The stress and negative affect model of adolescent alcohol use and the moderating effects of behavioral under-control. Journal of Studies on Alcohol.

[b12-arhw-20-3-175] Cooper ML, Peirce RS, Huselid RF (1994). Substance use and sexual risk taking among black adolescents and white adolescents. Health Psychology.

[b13-arhw-20-3-175] Curran PJ, Stice E, Chassin L The relation between adolescent alcohol use and peer alcohol use: A longitudinal random coefficients model. Developmental Psychology.

[b14-arhw-20-3-175] Deykin EY, Buka SL, Zeena TH (1992). Depressive illness among chemically dependent adolescents. American Journal of Psychiatry.

[b15-arhw-20-3-175] Donovan JE (1993). Young adult drinking-driving: Behavioral and psychosocial correlates. Journal of Studies on Alcohol.

[b16-arhw-20-3-175] Feldman SS, Elliott GR, Feldman SS, Elliott GR (1990). Capturing the adolescent experience. At the Threshold: The Developing Adolescent.

[b17-arhw-20-3-175] Grossman M, Chaloupka FJ, Saffer H, Laixuthai A (1994). Effects of alcohol price policy on youth: A summary of economic research. Journal of Research on Adolescence.

[b18-arhw-20-3-175] Holder HD, Zucker R, Boyd G, Howard J (1994). Commentary: Alcohol availability and accessibility as part of the puzzle: Thoughts on alcohol problems and young people. The Development of Alcohol Problems: Exploring the Biopsychosocial Matrix of Risk.

[b19-arhw-20-3-175] Hughes SO, Power TG, Francis DJ (1992). Defining patterns of drinking in adolescence: A cluster analytic approach. Journal of Studies on Alcohol.

[b20-arhw-20-3-175] Hussong AM, Chassin L (1994). The stress-negative affect model of adolescent alcohol use: Disaggregating negative affect. Journal of Studies on Alcohol.

[b21-arhw-20-3-175] Jacob T, Leonard K, Zucker R, Boyd G, Howard J (1994). Family and peer influences in the development of adolescent alcohol abuse. The Development of Alcohol Problems: Exploring the Biopsychosocial Matrix of Risk.

[b22-arhw-20-3-175] Jessor R, Jessor SL (1977). Personality and problem behavior. Problem Behavior and Psychosocial Development: A Longitudinal Study of Youth.

[b23-arhw-20-3-175] Johnston LD, O’Malley PM, Bachman JG (1995). Prevalence of drug use among eighth, tenth, and twelfth grade students. National Survey Results on Drug Use From the Monitoring the Future Study, 1975–1994: Volume 1. Secondary School Students.

[b24-arhw-20-3-175] Johnstone BM, Zucker R, Boyd G, Howard J (1994). Sociodemographic, environmental, and cultural influences on adolescent drinking behavior. The Development of Alcohol Problems: Exploring the Biopsychosocial Matrix of Risk.

[b25-arhw-20-3-175] Kaplan HB (1980). Antecedents of self-derogation. Deviant Behavior in Defense of Self.

[b26-arhw-20-3-175] Leigh BC, Stall R (1993). Substance use and risky sexual behavior for exposure to HIV: Issues in methodology, interpretation, and prevention. American Psychologist.

[b27-arhw-20-3-175] MacKinnon DP, Watson RR (1995). Review of the effects of the alcohol warning label. Alcohol, Cocaine, and Accidents: Drug and Alcohol Abuse Reviews 7.

[b28-arhw-20-3-175] Margulies RZ, Kessler RC, Kandel DB (1977). A longitudinal study of onset of drinking among high-school students. Journal of Studies on Alcohol.

[b29-arhw-20-3-175] Martin CS, Kaczynski NA, Maisto SA, Bukstein OM, Moss HB (1995). Patterns of DSM–IV alcohol abuse and dependence symptoms in adolescent drinkers. Journal of Studies on Alcohol.

[b30-arhw-20-3-175] McGue M, Sharma A, Benson P (1996). Parent and sibling influences on adolescent alcohol use and misuse: Evidence from a U.S. adoption cohort. Journal of Studies on Alcohol.

[b31-arhw-20-3-175] Moss HB, Kirisci L (1995). Aggressivity in adolescent alcohol abusers: Relationship with conduct disorder. Alcoholism: Clinical and Experimental Research.

[b32-arhw-20-3-175] Moss HB, Kirisci L, Gordon HW, Tarter RE (1994). A neuropsychologic profile of adolescent alcoholics. Alcoholism: Clinical and Experimental Research.

[b33-arhw-20-3-175] National Center for Statistics and Analysis (1992). 1991 Alcohol Fatal Crash Facts.

[b34-arhw-20-3-175] Newcomb MD, Bentler PM (1988). Consequences of Adolescent Drug Use: Impact on the Lives of Young Adults.

[b35-arhw-20-3-175] Pandina RJ, Johnson V, Labouvie EW, Glantz M, Pickens R (1992). Affectivity: A central mechanism in the development of drug dependence. Vulnerability to Drug Abuse.

[b36-arhw-20-3-175] Patterson GR, DeBaryshe BD, Ramsey E (1989). A developmental perspective on antisocial behavior. American Psychologist.

[b37-arhw-20-3-175] Robins LN, Pryzbeck TR, Jones CL, Battjes RJ (1985). Age of onset of drug use as a factor in drug and other disorders. Etiology of Drug Abuse: Implications for Prevention.

[b38-arhw-20-3-175] Rowe DC, Gulley BL (1992). Sibling effects on substance use and delinquency. Criminology.

[b39-arhw-20-3-175] Single E, Zucker R, Boyd G, Howard J (1994). The impact of social and regulatory policy on drinking behavior. The Development of Alcohol Problems: Exploring the Biopsychosocial Matrix of Risk.

[b40-arhw-20-3-175] Smith GT, Goldman MS, Greenbaum PE, Christiansen BA (1995). Expectancy for social facilitation from drinking: The divergent paths of high-expectancy and low-expectancy adolescents. Journal of Abnormal Psychology.

[b41-arhw-20-3-175] U.S. Department of Health and Human Services (1991). Healthy People 2000: National Health Promotion and Disease Prevention Objectives.

[b42-arhw-20-3-175] Windle M, Miller-Tutzauer C, Domenico D (1992). Alcohol use, suicidal behavior, and risky activities among adolescents. Journal of Research on Adolescence.

[b43-arhw-20-3-175] Yamaguchi K, Kandel DB (1984). Patterns of drug use from adolescence to young adulthood: III. Predictors of progression. American Journal of Public Health.

